# Supplementary material for: In silico Derivation of HLA-Specific Alloreactivity Potential from Whole Exome Sequencing of Stem-Cell Transplant Donors and Recipients: Understanding the Quantitative Immunobiology of Allogeneic Transplantation
Source: Front Immunol. 2014 Nov 6;5:529. doi: 10.3389/fimmu.2014.00529 (PMC4222229; doi:10.3389/fimmu.2014.00529)
Supplement: Supplementary file 1 [file Table_1.DOCX]

**Supplementary Table 1.** SMM versus MHC binding affinities of various peptides for HLA-B1503 in a single patient (patient 4).

| HLA Allele | Gene Name | Gene ID | Chromosome | Peptide sequence | IC50 SMM | IC50 MHC |
| --- | --- | --- | --- | --- | --- | --- |
| HLA-B*15:03 | DSEL | NM_032160 | chr18 | FQWCFYLSF | 0.21 | 2.42 |
| HLA-B*15:03 | KIAA0141 | NM_001142603 | chr5 | FQLSVSITF | 0.69 | 2.02 |
| HLA-B*15:03 | OR5AU1 | NM_001004731 | chr14 | LQRLLFMVF | 0.78 | 3.02 |
| HLA-B*15:03 | DCHS1 | NM_003737 | chr11 | FQRPHYVAF | 0.82 | 1.91 |
| HLA-B*15:03 | SLC22A6 | NM_153278 | chr11 | LQLLVSVPF | 0.86 | 3.40 |
| HLA-B*15:03 | ZNF106 | NM_022473 | chr15 | LQITTCPTF | 1.09 | 2.60 |
| HLA-B*15:03 | OR10P1 | NM_206899 | chr12 | SMMTATIVF | 1.14 | 2.26 |
| HLA-B*15:03 | DNAH14 | NM_001373 | chr1 | SQSKLTSTF | 1.41 | 1.88 |
| HLA-B*15:03 | TARBP1 | NM_005646 | chr1 | MKFGTNAYM | 1.47 | 12.26 |
| HLA-B*15:03 | PREPL | NM_001171603 | chr2 | LKKYHLTKF | 1.48 | 25.40 |
| HLA-B*15:03 | CYP1B1 | NM_000104 | chr2 | LKWPNPENF | 1.53 | 21.21 |
| HLA-B*15:03 | OR2T8 | NM_001005522 | chr1 | LQAVVTLSF | 1.71 | 2.10 |
| HLA-B*15:03 | ASB18 | NM_212556 | chr2 | WQVKSPTTF | 1.72 | 1.97 |
| HLA-B*15:03 | EPX | NM_000502 | chr17 | SQVPLSSAF | 1.75 | 1.85 |
| HLA-B*15:03 | ADAM2 | NM_001464 | chr8 | LMNAIFVSF | 1.75 | 3.10 |
| HLA-B*15:03 | PKP1 | NM_001005337 | chr1 | RHFSSYSQM | 1.98 | 16.71 |
| HLA-B*15:03 | CAPN14 | NM_001145122 | chr2 | RQNEFFTKF | 2.12 | 1.95 |
| HLA-B*15:03 | SH3D19 | NM_001128923 | chr4 | YMHGDVLVM | 2.20 | 2.90 |
| HLA-B*15:03 | ATXN1 | NM_001128164 | chr6 | KMGLSAAPF | 2.42 | 6.55 |
| HLA-B*15:03 | ABP1 | NM_001272072 | chr7 | FAFRLRSSF | 2.49 | 2.77 |
| HLA-B*15:03 | MUC16 | NM_024690 | chr19 | SKHASHSTI | 2.58 | 98.91 |
| HLA-B*15:03 | KIAA0226 | NM_001145642 | chr3 | AKSSSSNLF | 2.68 | 6.05 |
| HLA-B*15:03 | MYO15A | NM_016239 | chr17 | LQVLRAYSF | 2.72 | 3.39 |
| HLA-B*15:03 | SVIL | NM_003174 | chr10 | AKHLWNGSF | 2.74 | 9.85 |
| HLA-B*15:03 | OR8D4 | NM_001005197 | chr11 | RQRHTPMYY | 2.86 | 6.02 |
| HLA-B*15:03 | GPAM | NM_001244949 | chr10 | IMSTHIVAF | 2.93 | 2.55 |
| HLA-B*15:03 | SERPINB4 | NM_002974 | chr18 | RKSKESNIF | 2.97 | 10.77 |
| HLA-B*15:03 | MUC16 | NM_024690 | chr19 | LQSLLGPMF | 3.18 | 4.69 |
| HLA-B*15:03 | OR1S1 | NM_001004458 | chr11 | LKLSCSDTM | 3.27 | 25.72 |
| HLA-B*15:03 | APOBEC3B | NM_001270411 | chr22 | YKCFQLTWF | 3.48 | 37.91 |
| HLA-B*15:03 | C8A | NM_000562 | chr1 | RKAQCGQDF | 3.48 | 16.82 |
| HLA-B*15:03 | OR4D2 | NM_001004707 | chr17 | LQRFLFIMF | 3.52 | 7.07 |
