# Supplementary material for: In silico Derivation of HLA-Specific Alloreactivity Potential from Whole Exome Sequencing of Stem-Cell Transplant Donors and Recipients: Understanding the Quantitative Immunobiology of Allogeneic Transplantation
Source: Front Immunol. 2014 Nov 6;5:529. doi: 10.3389/fimmu.2014.00529 (PMC4222229; doi:10.3389/fimmu.2014.00529)
Supplement: Supplementary file 2 [file Table_2.DOCX]

**Supplementary Table 2:** Tissue Gene Expression for Presented Peptides (IC50 <500nM). Number of genes for which the relative expression unit value is >10.

|  | **Adipose** | **Colon** | **Kidney** | **Leukocyte** | **Liver** | **Lung** | **Lymph**  **Node** | **Skeletal**  **Muscle** |
| --- | --- | --- | --- | --- | --- | --- | --- | --- |
| P2 | 110 | 87 | 94 | 109 | 82 | 105 | 110 | 68 |
| P3 | 212 | 194 | 213 | 238 | 153 | 257 | 227 | 141 |
| P4 | 211 | 205 | 266 | 258 | 144 | 259 | 256 | 172 |
| P5 | 284 | 258 | 323 | 314 | 215 | 319 | 275 | 231 |
| P7 | 325 | 251 | 389 | 397 | 245 | 391 | 353 | 287 |
| P8 | 284 | 233 | 291 | 364 | 231 | 326 | 313 | 289 |
| P10 | 238 | 219 | 273 | 260 | 184 | 269 | 255 | 192 |
| P16 | 27 | 30 | 33 | 28 | 29 | 39 | 28 | 28 |
| P23 | 59 | 42 | 69 | 69 | 47 | 67 | 53 | 49 |
