# Supplementary figures and images for: In silico Derivation of HLA-Specific Alloreactivity Potential from Whole Exome Sequencing of Stem-Cell Transplant Donors and Recipients: Understanding the Quantitative Immunobiology of Allogeneic Transplantation
Source: Front Immunol. 2014 Nov 6;5:529. doi: 10.3389/fimmu.2014.00529 (PMC4222229; doi:10.3389/fimmu.2014.00529)

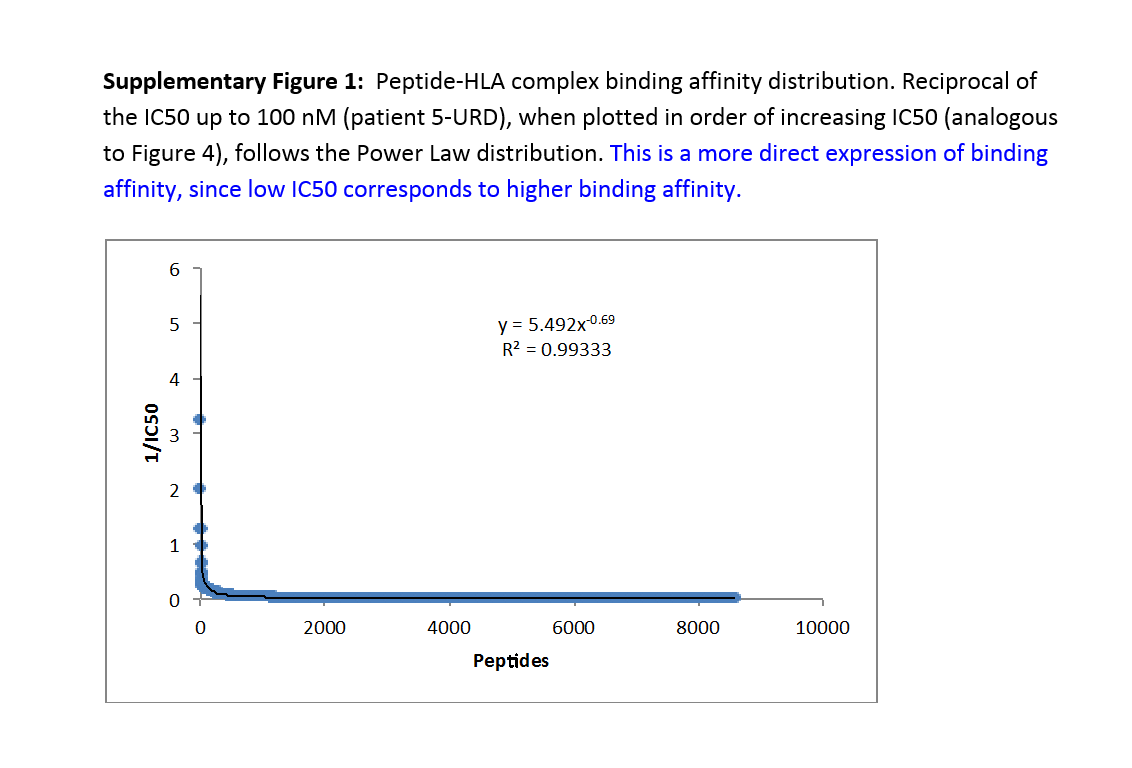

Supplement: Supplementary file 3 [file Image_1.TIF]

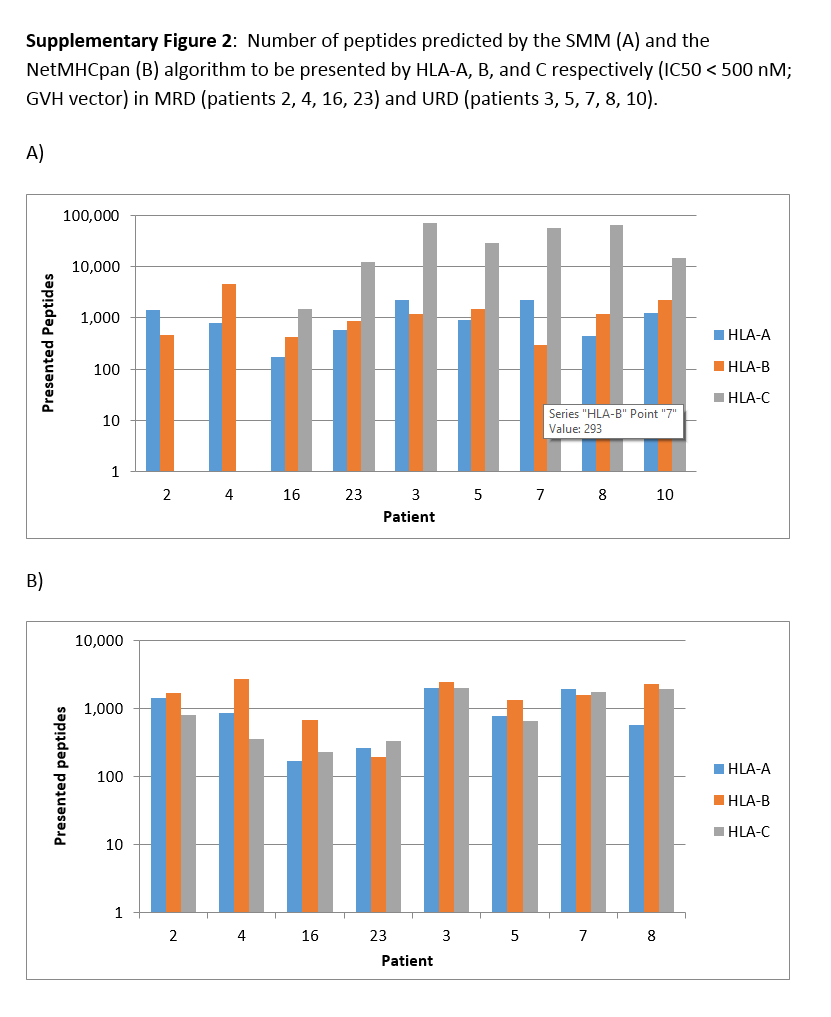

Supplement: Supplementary file 4 [file Image_2.TIF]
